# Exome sequencing identifies 2 novel presenilin 1 mutations (p.L166V and p.S230R) in British early-onset Alzheimer's disease^☆^

**DOI:** 10.1016/j.neurobiolaging.2014.04.026

**Published:** 2014-10

**Authors:** Celeste Sassi, Rita Guerreiro, Raphael Gibbs, Jinhui Ding, Michelle K. Lupton, Claire Troakes, Katie Lunnon, Safa Al-Sarraj, Kristelle S. Brown, Chirstopher Medway, Jenny Lord, James Turton, David Mann, Julie Snowden, David Neary, Jeniffer Harris, Jose Bras, ARUK Consortium, Kevin Morgan, John F. Powell, Andrew Singleton, John Hardy

**Affiliations:** a University College London (UCL) Institute of Neurology, London, UK; b Laboratory of Neurogenetics, National Institute on Aging, National Institutes of Health, Bethesda, MD, USA; c Institute of Psychiatry, King's College London, London, UK; d School of Molecular Medical Sciences, Institute of Genetics, Queen's Medical Centre, University of Nottingham, Nottingham, UK; e Institute of Brain, Behaviour, and Mental Health, The University of Manchester, Manchester, UK; f Cerebral Function Unit Greater Manchester Neuroscience Centre, Manchester, UK

**Keywords:** Early-onset Alzheimer's disease, *APP*, *PSEN1*, *PSEN2*, British cohort

## Abstract

Early-onset Alzheimer's disease (EOAD) represents 1%–2% of the Alzheimer's disease (AD) cases, and it is generally characterized by a positive family history and a rapidly progressive symptomatology. Rare coding and fully penetrant variants in amyloid precursor protein (*APP*), presenilin 1 (*PSEN1*), and presenilin 2 (*PSEN2*) are the only causative mutations reported for autosomal dominant AD. Thus, in this study we used exome sequencing data to rapidly screen rare coding variability in APP, *PSEN1*, and *PSEN2*, in a British cohort composed of 47 unrelated EOAD cases and 179 elderly controls, neuropathologically proven. We report 2 novel and likely pathogenic variants in *PSEN1* (p.L166V and p.S230R). A comprehensive catalog of rare pathogenic variants in the AD Mendelian genes is pivotal for a premortem diagnosis of autosomal dominant EOAD and for the differential diagnosis with other early onset dementias such as frontotemporal dementia (FTD) and Creutzfeldt-Jakob disease (CJD).

## 1 Introduction

Early-onset Alzheimer's disease (EOAD), with onset of symptoms before 65 years of age, accounts for 1%–2% of the Alzheimer's disease (AD) cases. The disease commonly clusters within families and presents a rapid and severe progression. Generally, EOAD is a polygenic and complex disease. On the contrary, 10% of EOAD cases present a Mendelian autosomal dominant pattern of inheritance and are caused by rare and fully penetrant mutations in amyloid precursor protein (*APP*), presenilin 1 (*PSEN1*), and presenilin 2 (*PSEN2*), leading to Aß_42_ overproduction (Goate et al., 1991; Pastor and Goate, 2004; Raux et al., 2005; Sherrington et al., 1995). *PSEN1* is the most common cause of EOAD (18%–50%), with 185 mutations reported (www.molgen.database/). By contrast, *APP* and *PSEN2* have been described in 5% and <1% of the EOAD cases, respectively (www.molgen.database/). Given the pivotal role of these genes in AD pathogenesis, a detailed catalog of pathogenic variants and an algorithm for their classification are fundamental premises for an effective genetic screening of the patients.

## 2 Methods

### 2.1 Cases and controls

Our cohort was composed of 47 independent EOAD (age at onset ≤65 years) autopsy proven cases and 179 elderly (>60 years), unrelated controls. Three patients reported a positive family history of AD. The other patients were referred as apparently sporadic EOAD cases.

All the patients and controls were Caucasian, mostly from the UK (London, Manchester, Nottingham, and Edinburgh) and to a lesser extent from North America. The average age at diagnosis was 57.7 years (range 41–64 years) for the EOAD patients and the mean age of ascertainment was 78 years (range 60–102 years) for the controls (Table 1).

Written informed consent was obtained for each individual and the study was approved by the appropriate institutional review boards.

### 2.2 Exome sequencing

Library preparation for next-generation sequencing was performed according to the NimbleGen (Roche NimbleGen v2) and TruSeq (Illumina) sample-preparation protocols. DNA libraries were then hybridized to exome-capture probes with NimbleGen SeqCap EZ Human Exome Library, version 2.0 (Roche NimbleGen) or TruSeq (Illumina). Each capture method covers the *APP*, *PSEN1*, and *PSEN2* loci. Exome-enriched libraries were sequenced on the Illumina HiSeq 2000 using 2 × 100 bp paired end read cycles.

### 2.3 Bioinformatics

Sequence alignment and variant calling was performed against the reference human genome (UCSC hg19). Paired end sequence reads (2 × 100 bp paired end read cycles) were aligned using the Burrows-Wheeler aligner (Li and Durbin, 2009). Format conversion and indexing were performed with Picard (www.picard.sourceforge.net/index.shtml). The Genome Analysis Toolkit was used to recalibrate base quality scores, perform local re-alignments around indels, and to call and filter the variants (McKenna et al., 2010). VCFtools was used to annotate gene information for the remaining novel variants. In total, 450,987 single-nucleotide variants and 41,678 indels were called. We used snpEff v3.2 software to annotate the variants. Variants were checked against established databases (1000 Genomes Project and dbSNP v.134). The protein coding effects of variants was predicted using SIFT, Polyphen2, and SeattleSeq Annotation (gvs.gs.washington.edu/SeattleSeqAnnotation). All variants within the coding regions of *APP*, *PSEN1*, and *PSEN2* were annotated for both cases and controls.

### 2.4 Sanger sequencing

All rare variants identified by whole-exome sequencing in the candidate genes were validated by Sanger sequencing.

Primers for exons harboring rare variants were designed in Primer3 (http://bioinfo.ut.ee/primer3-0.4.0/) using UCSC (http://genome.ucsc.edu/) reference sequences NM_000484 (*APP*), NM_000021.3 (*PSEN1*), and NM_000447.2 (*PSEN2*).

Purified sequences were analyzed on an ABI 3730 DNA Analyzer (Applied Biosystems, CA, USA) and electropherograms were visualized in Sequencher software (version 4.2 Gene Codes Corporation, MI, USA).

### 2.5 Apoe genotyping

*APOE* genotypes comprising the *APOE* ɛ2, ɛ3, and ɛ4 alleles were assayed using the TaqMan method (Applied Biosystems Inc [ABI], Foster City, CA, USA). Single nucleotide polymorphism– specific primers and probes were designed by ABI (TaqMan genotyping assays).

## 3 Results

We report the experience of a mutation screening in *APP*, *PSEN1*, and *PSEN2* in 47 EOAD unrelated cases and 179 elderly controls neuropathologically proven, from the UK. 9 EOAD cases (19%) and 5 controls (2.8%) carried at least 1 rare coding variant in the genes studied.

In our EOAD cohort, we identified a total of 8 rare coding variants in *APP*, *PSEN1*, and *PSEN2*, absent in controls. Of these, 3 have been previously reported in familial AD (p.C410Y and p.113+1delG in *PSEN1* and p.V717L in *APP*) (Campion et al., 1995; De Jonghe et al., 2001; Murrell et al., 2000; Tysoe et al., 1998) and 4 are novel variants: 2 detected in *PSEN1* (p.L166V and p.S230R) and 2 in *APP* (p.K496Q and p.620L). In addition, we report a rare and likely tolerated polymorphism in *PSEN2* (p.S130L, rs63750197), present in 1 case and 1 control (Table 2).

*PSEN1* p.L166V maps to the third transmembrane domain (TM3) of PS1, on the α-helix surface and corresponds to a conserved domain in *PSEN2* (p.L172), where no variants or mutations have been reported. Moreover, it has been described as possibly damaging by in silico predictions (SIFT and Polyphen2). This variant was detected in a very early onset case, presenting typical and rapidly progressing AD features with extrapyramidal signs (parkinsonism and myoclonus). At the age of 42 years, this patient developed difficulties in memory, necessitating his retirement from work. Over the next 2 years, he presented a decline in cognitive function, culminating in his medical referral. His memory loss had become more severe, he had difficulties in verbal expression, reading, writing, and calculation, and he was spatially disoriented. He had become generally apathetic. Neurologic examination revealed extrapyramidal signs and myoclonus. Neuropsychological examination revealed profound impairments in memory, language, perceptuospatial skills, and praxis. Social skills were well preserved, and he was frustrated by his difficulties.

He was reviewed at regular intervals over the following 4 years. During that time there was dramatic decline in his physical and cognitive skills. He showed marked parkinsonism and myoclonus. Speech was unintelligible, and he showed profound perceptual and spatial impairments. A single photon emission computerized tomography (SPECT) scan, 4 years after symptom onset, showed temporoparietal hypoperfusion. The profile remained typical of Alzheimer's disease. He died aged 50 years, 8 years after onset of symptoms. The neuropathological examination revealed advanced AD lesions (Braak VI, CERAD C). There was no relevant previous medical history and no clear history of dementia in any first-degree relative, still alive, and in good health conditions.

Finally, the *PSEN1* p.L166V was not found in 179 healthy elderly neuropathologically confirmed controls, or in any control present in genomic variant databases (dbSNP v.137, ESP).

*PSEN1* p.S230R is a nonconservative amino acid change in the fifth transmembrane domain (TM5), on the α-helix surface of PS1. It corresponds to a conserved residue in *PSEN2* (p.S236), where no variants or mutations have been described and was predicted as possibly damaging by SIFT and Polyphen2. The carrier presented features consistent with the reported clinical phenotype in most *PSEN1* mutations. The patient was diagnosed at 58 years, with a 2-year history of problems in memory. Neurologic examination was entirely normal. His behavior was socially appropriate and he was insightful into his difficulties. Neuropsychological examination confirmed the problems in memory and language. Performance in other cognitive domains was well preserved. Functional imaging using SPECT showed left posterior hypoperfusion. The patient's father had died in his early 60s of a similar dementing illness. The patient was clinically diagnosed with Alzheimer's disease, which was suspected to be familial.

He was reviewed at regular intervals. His cognitive skills deteriorated gradually. His memory became worse; he had increased difficulties in verbal expression and visuospatial tasks. He presented parkinsonian signs and myoclonus. A repeat SPECT scan revealed bilateral parietal hypoperfusion, more marked on the left side. Latterly, he became severely parkinsonian and mute. He died aged 66 years, 10 years after onset of symptoms. The neuropathological features revealed a fully developed Alzheimer's disease (Braak VI, CERAD C).

Finally, although we cannot rule out a pathogenic role for *APP* p.K496Q and p.620L, we suggest they are likely to be rare benign polymorphisms, as they cluster outside exon 16 and 17, where all pathogenic mutations have been reported up to date (www.molgen.database/).

## 4 Discussion

Mutations in *APP*, *PSEN1*, and *PSEN2* are the only fully penetrant variants described for EOAD. Thus, in this study we used exome-sequencing data to investigate rare coding variability in *APP*, *PSEN1*, and *PSEN2*, in a British cohort composed of 47 unrelated EOAD cases and 179 elderly controls. In this cohort, 6 patients of 47 (12.7%) and none of the controls carried a rare coding variant in *PSEN1*. This confirms that *PSEN1* is the major gene involved in EOAD and suggests that rare coding variability in this gene is commonly detrimental. Among the variants detected in *PSEN1*, 2 are novel (p.L166V and p.S230R), likely harmful and classified as probable pathogenic, following the algorithm proposed by Guerreiro et al. (2010). These variants have been detected in 2 patients who presented the typical hallmarks of *PSEN1* mutations: early onset of symptoms, aggressive symptomatology, and extrapyramidal signs (parkinsonism and myoclonus).

*PSEN1* p.L166 codon seems to be a very vulnerable site for mutations: 4 different causal mutations (p.L166del, p.L166H, p.L166P, and p.L166R) have been previously described in this same residue in 3 familial AD cases (p.L166R, p.L166P, and p.L166del) (Ezquerra et al., 2000; Knight et al., 2007; Moehlmann et al., 2002) and in 1 pseudo-sporadic case (p.L166H) (Pantieri et al., 2005). From these, p.L166P is known to be a very aggressive mutation: it causes onset of symptoms before the third decade of age, significantly increases Aβ_42_ production, and impairs NOTCH1 signaling in a cell culture system (Moehlmann et al., 2002). Furthermore, the amino acid position 166, located in the TM3 of PS1 shows a significant phylogenetic conservation across different species and in homologous proteins such as PS2, suggesting that the position is of functional significance.

In our cohort, the *PSEN1* p.L166V variant has been detected in a patient with very young onset of age (42 years at diagnosis), thus supporting the severe effects of an amino acid substitution in this conserved residue. Surprisingly, the patient's parents were not affected by Alzheimer's disease. This suggests that the p.L166V may be a de novo mutation or that other genetic factors may influence the phenotype of *PSEN1* mutations.

*PSEN1* p.S230R clusters in the TM5 domain of PS1 and is the homologous of a conserved residue in *PSEN2* (p.S236). The patient carrying this variant presented a family history of AD. Recently, a different missense mutation in this codon (p.S230I) has been reported as possible pathogenic in a French family with EOAD (Wallon et al., 2012). Thus, the p.S230R is likely to be a novel mutation for early onset, autosomal dominant Alzheimer's disease.

In summary, we confirm that *PSEN1* is commonly associated with EOAD and we report 2 novel and likely pathogenic mutations detected in this gene (p.L166V and p.S230R). A comprehensive catalog of rare pathogenic variants in Alzheimer's disease Mendelian genes is pivotal: (1) for a premortem diagnosis of EOAD patients; (2) for the differential diagnosis of other types of early onset dementias (FTD and CJD); and (3) for the identification of at risk relatives who may be potential candidates for clinical trials.

## Disclosure statement

The authors have nothing to disclose.

## Figures and Tables

**Table 1:** Cohort

Cohort	n	Diagnosis	Sequencing strategy	Age (y) mean ± SD (range)	Male (%)	*APOE* e4+ (%)
EOAD	47	Clinical and neuropathological	Exome sequencing	57.7 (41–64)	55	57
Controls	179	Clinical and neuropathological	Exome sequencing	78 (60–102)	55	40

Key: EOAD, early-onset Alzheimer's disease; SD, standard deviation.

**Table 2:** Rare coding variants found in *APP*, *PSEN1*, and *PSEN2* in 47 EOAD cases and 179 elderly controls. *APP* (NM_000484); presenilins 1 and 2, *PSEN1* (NM_000021.3), and *PSEN2* (NM_000447.2)

Variant interpretation	Gene	Variant	Minor allele	Status	SIFT and/or polyphen	EOAD (n = 47)					Controls (n = 179)		
						Count (%)	(AAO-AAD) (y)	Genotype	*APOE*	Comment	Count (%)	Genotype	*APOE*
Known causative mutations													
	*APP*	p.V717L	A	rs63750264	Possibly damaging	1 (2)	59	C/A	ε3ε3	Causal mutation in FAD	0	—	—
	*PSEN1*	p.C410Y	A	rs661	Possibly damaging	1 (2)	49–57	G/A	ε3ε3	Causal mutation in FAD	0	—	—
	*PSEN1*	p.113 + 1delG, splice5	delG	rs63751475	Possibly damaging	2 (4.2)	41–51	G/-	ε4ε4, ε3ε3	Causal mutation in FAD	0	—	—
Probable pathogenic variants^a^													
	*PSEN1*	p.L166V	G	Novel	Possibly damaging	1 (2)	42–50	C/G	ε3ε3	L166del, L166P, L166 R causal mutations in FAD; L166H causal mutation in a pseudo-sporadic AD case	0	—	—
	*PSEN1*	p.S230R	G	Novel	Possibly damaging	1 (2)	56–66	T/G	ε3ε3	S230I reported in a French EOAD family	0	—	—
Likely rare benign polymorphisms													
	*APP*	p.K496Q	G	Novel	Possibly damaging	1 (2)	NA	T/G	ε3ε4	Exon 11	0	—	—
	*APP*	p.620L	A	Novel	Tolerated	1 (2)	65	G/A	ε3ε4	Exon 14	0	—	—
	*PSEN2*	p.S130L	T	rs63750197	Possibly damaging	1 (2)	61–65	C/T	ε3ε3		1 (0.5)	C/T	ε2ε2

Key: AAD, age at death; AAO, age at onset; AD, Alzheimer's disease; APP, amyloid precursor protein; FAD, familial Alzheimer's disease; PSEN1, presenilin 1; PSEN2, presenilin 2.

a Classification based on the algorithm proposed by Guerreiro et al. (2010).
